# Transplantation of Induced Pluripotent Stem Cells Improves Functional Recovery in Huntington's Disease Rat Model

**DOI:** 10.1371/journal.pone.0101185

**Published:** 2014-07-23

**Authors:** Shuhua Mu, Jiachuan Wang, Guangqian Zhou, Wenda Peng, Zhendan He, Zhenfu Zhao, CuiPing Mo, Junle Qu, Jian Zhang

**Affiliations:** 1 College of Optoelectronics Engineering, Shenzhen University, Shenzhen, China; 2 School of Medicine, Shenzhen University, Shenzhen, China; University of Central Florida, United States of America

## Abstract

The purpose of this study was to determine the functional recovery of the transplanted induced pluripotent stem cells in a rat model of Huntington's disease with use of ^18^F-FDG microPET/CT imaging.

**Methods:**

In a quinolinic acid-induced rat model of striatal degeneration, induced pluripotent stem cells were transplanted into the ipsilateral lateral ventricle ten days after the quinolinic acid injection. The response to the treatment was evaluated by serial ^18^F-FDG PET/CT scans and Morris water maze test. Histological analyses and Western blotting were performed six weeks after stem cell transplantation.

**Results:**

After induced pluripotent stem cells transplantation, higher ^18^F-FDG accumulation in the injured striatum was observed during the 4 to 6-weeks period compared with the quinolinic acid-injected group, suggesting the metabolic recovery of injured striatum. The induced pluripotent stem cells transplantation improved learning and memory function (and striatal atrophy) of the rat in six week in the comparison with the quinolinic acid-treated controls. In addition, immunohistochemical analysis demonstrated that transplanted stem cells survived and migrated into the lesioned area in striatum, and most of the stem cells expressed protein markers of neurons and glial cells.

**Conclusion:**

Our findings show that induced pluripotent stem cells can survive, differentiate to functional neurons and improve partial striatal function and metabolism after implantation in a rat Huntington's disease model.

## Introduction

Huntington's disease (HD) is characterized by the expansion of CAG repeats in the huntingtin gene and the loss of medium spiny neurons in the striatum, resulting in progressive cognitive impairment, neuropsychiatric symptoms, and involuntary choreiform movements [1]. The neuropathological changes in HD are selective and progressive degeneration of striatal GABAergic medium spiny projection neurons [2]. Intrastriatal injection of an excitotoxin such as quinolinic acid (QA) mimics some of the pathology of HD, including the loss of projection GABAergic neurons with a relative preservation of interneurons, and allows for the study of therapeutics, such as transplantation [3], [4]. Currently, there is no proven medical therapy to alleviate the onset or progression of Huntington's disease [5].

The clinical uses of cell replacement therapy in neurodegenerative diseases have been investigated for the last 20 years. Although the procedures are theoretically feasible, some limitations of the therapy still give cause for concern [6]. The transplantation of fetal striatal tissue to the striatum to modify HD progression in humans has been investigated, and some favorable effects have been found [7], [8], but it does not alter the toxic effects of mutant huntingtin and has difficulties in tissue availability and viability, high risk of rejection, ethical arguments and concerns about contamination and heterogeneity of the tissues [9].

One solution to above problems may be to use induced pluripotent stem cells (iPSCs). The projected use of iPSC derivative cell types in cell-based therapies offers unique advantages over the use of many adult stem cell types with respect to limited proliferation capacity and donor availability, and where patient matched cells may overcome the vexing issues of immune rejection associated with human cell transplantation treatments [6], [10]. iPSCs can be generated by transduction of defined transcription factors from adult somatic cells through reprogramming and have been differentiated in vitro into the early neural stem cell stage or the neural lineage, including neurons and glial cells [11]. More recently, iPSCs have been applied to a variety of nervous system disease, including stroke [11], [12], spinal cord injury [13], [14] and Parkinson disease [15], [16]. However, to better understand the in vivo behavior and efficacy of iPSCs, a noninvasive, sensitive, and clinically applicable approach for tracking the transplanted iPSCs and monitoring the therapeutic response in living subjects needs to be developed.

PET is one of the best-suited modalities to evaluate stem cell therapy, since it can be used in patients clinically for both cell trafficking and monitoring the response to therapy [17], . PET studies of patients with HD demonstrate a dramatic loss of striatal glucose metabolism, even in presymptomatic stages [19], [20]. PET can also be used to detect the subtle changes of glucose metabolism in vivo after stem cell therapy in various neurologic disease models, including traumatic brain injury [21], Parkinson disease [22], and Huntington disease [4]. To highly precise measurement of the FDG uptake in injured striatum, PET imaging in combination with contrast-enhanced CT was used in this study.

The aims of the present study were to investigate whether transplanted iPSC migrated and survived in QA-injured striatum of rats, improving functional and metabolic deficits of striatum, and whether ^18^F-FDG PET imaging can monitor the improvement of cerebral energy metabolism in the striatum of rat model of HD.

## Materials and Methods

### 1. Experimental design and animal groups

All animal procedures were performed according to the National Institutes of Health Guide for the Care and Use of Laboratory Animals and were approved by the Guangdong Medical Laboratory Animal Center Institutional Animal Care and Use Committee. Sprague-Dawley rats were housed with a 12-h light/dark cycle and *ad libitum* access to food and water. 24 adult Sprague-Dawley male rats (body weight, 250–280 g) were randomly assigned to one of the following three experimental groups (eitht per group): the iPSC transplantation group (rats received both QA injection and iPSC transplantation, QA+iPSC), the QA injection group (rats received both QA injection and PBS transplantation, QA+PBS), and the control group (rats received only saline injection). The stem cell transplantation or PBS injection was performed 10 d after QA lesions. The Morris water maze task was performed at 5 weeks after cell transplantation. ^18^F-FDG small-animal PET/CT scans were performed before stem cell transplantation and at weeks 1, 2, 4, and 6 after cell transplantation. Animals were then sacrificed for histological, immunohistochemical (n = 4) and Western blot (n = 4) analysis.

### 2. Striatal quinolinic acid lesions

Unilateral lesions of the left striatum were achieved by intrastriatal injection of QA. Rats were anesthetized with a solution of ketamine (75 mg/kg) and xylazine (12.5 mg/kg). QA (Sigma) injections were made with the help of a Kopf stereotactic apparatus. Each rat was injected with 100 nmol of QA dissolved in 1 µl of saline into the left striatum at the following coordinates: 1.2 mm anterior, 2.5 mm lateral to bregma, and 5.0 mm below the dura surface. The control group received only vehicle. Injections were performed with a Hamilton syringe. The liquid was injected over a 5-min period, after which the needle was left in place for additional 15 min and then slowly removed. After surgery, rats were allowed to recover for at least 10 days before stem cell transplantation. There is no immune depressant being used during the experiment.

### 3. Stem Cell Preparation

Enhanced green fluorescent protein (EGFP)–labeled mouse iPSCs was bought from SiDan Sai Biotechnology Co., Ltd (China). The iPSCs was derived from the fibroblast of C57BL/6 mouse and was engineered to express green fluorescent protein (GFP) via stable transfection with a lentivirus construct containing EGFP gene under the control of the constitutive promoter from the elongation factor 1α. The iPSCs was cultured as described previously [10]. Briefly, mouse iPSCs was maintained on a mitotically inactivated mouse embryonic fibroblast feeder layer in knockout Dulbecco modified Eagle medium (Invitrogen) containing 10% fetal bovine serum (Invitrogen), 10% knockout serum replacement (Invitrogen), 2 mML-glutamine (Invitrogen), ×100 nonessential amino acids (Invitrogen), ×1000 β-2-mercaptoethanol (Invitrogen), 50 units of penicillin and a 50 mg/mL dose of streptomycin (Invitrogen), and mouse leukemia inhibitory factor (Invitrogen). Before stem cell transplantation, iPSC colonies were passaged up to 4 times without feeder cells on 60-mm culture dishes coated with 0.1% gelatin to eliminate contamination of the mouse embryonic fibroblasts.

### 4. Transplantation Procedure

Rats were anesthetized with a solution of ketamine (75 mg/kg) and xylazine (12.5 mg/kg) and placed in a stereotactic instrument. About 1.0×10^6^ suspended iPSCs or PBS was stereotactically injected into the left lateral ventricle (0.92 mm anterior to the bregma, 1.2 mm lateral to the midline, and 3.1 mm beneath the dura) in a volume of 20 µl over 10 min with the use of a Hamilton microsyringe. The needle was left in place for an additional 10 min and then removed slowly. All surgical procedures were conducted under aseptic conditions. There is no immune depressant being used during the exprement.

### 5. Morris water maze task

As there is evidence that huntington's disease in rodents cause impairments when animals are tested in the Morris water maze [23], we used the Morris water maze for the current experiments. The Morris water maze task was performed according to our previous study [24] and the other [25]. Briefly, the rats were trained for 5 consecutive days, followed by the probe trial on day 6. The rats were let down in four random places (N, S, E, W) in the pool. The order of these was changed daily in a random manner. The rats were trained four times per day (120 sec/trial or until they found the platform). After the 120-sec swim, they were allowed to stay on the platform for 30 sec before the next swim trial. Single probe trials to test reference memory were conducted 1 day after the last training session. Rats were released at a random start position, and were allowed to swim during 120 sec in the absence of the platform. The tracks were recorded using video camera and Ethovision software (Noldus). Owing to different swim speeds in the different groups, the latencies of training days 1–5 were compared with the average latency for each day. For the analysis of the probe tests, the number of target annulus crossovers was compared.

### 6. MicroPET/CT scans and Image analysis

Lesion-induced deficits and changes after transplantation were assessed in vivo using microPET/CT scanning of [^18^F] fluorodeoxyglucose (^18^F-FDG) uptake to image metabolic activity, as described previously [26]. Briefly, for the first set of animals, 10 days after striatal lesion as well as 1, 2, 4 and 6 weeks after transplantation, rats were injected with 450 µCi of ^18^F-FDG in the tail vein, in a maximum volume of 0.5 ml of sterile saline. After injection, they were returned to their cages for a 45-min uptake period in a dark and quiet environment. After the uptake period each animal was anesthetized with a solution of ketamine (75 mg/kg) and xylazine (12.5 mg/kg). The PET data were acquired at 60 min after intravenous injection in 3-dimensional mode, with emission scans of 10 min per bed position. Imaging started with a low-dose CT scan (30 mA), immediately followed by a PET scan. The CT scan was used for attenuation correction and localization of the lesion site. The coronal, transaxial, and sagittal views of PET imaging and MIP (maximum intensity projection) of the model rats were obtained after image reconstruction with a slice thickness of two-micrometer. The average radioactivity concentration within the lesion area was obtained from the mean pixel values. The lesion-to-normal homologous contralateral ratio was used for semiquantitative analysis.

### 7. Histology and Immunohistochemistry

Animals were anesthetized with a solution of ketamine (75 mg/kg) and xylazine (12.5 mg/kg) and perfused first with 400 ml of saline and then 400 ml of 4% paraformaldehyde (in 0.1 M phosphate buffer, pH 7.4). Brains were then removed and postfixed in the same fixative, and then coronal sections (30 µm) were cut on a vibratome (VIBRATOME, #053746). Sections were stained with Nissl according to conventional staining methods.

For immunohistochemistry, sections were pretreated with 0.3% H_2_O_2_ in 0.01 M PBS at 37°C for 30 min. To carry out conventional single-label immunohistochemistry, separate series of sections were incubated overnight at 4°C in mouse anti-neuronal nuclei (NeuN) (1∶1000, Millipore), rabbit anti-Dopamine- and cAMP-regulated neuronal phosphoprotein (Darpp32) (1∶500, Millipore), mouse anti-glial fibrillary acidic protein (GFAP) (1∶1000, Santa Cruz Biotechnology), and rabbit anti- ionized calcium binding adaptor molecule 1 (Iba-1) (1∶1000, Millipore). Sections were then rinsed and incubated in anti-mouse IgG or anti-rabbit IgG (1∶200, Sigma), followed by incubating in the appropriate mouse or rabbit PAP complex (1∶200, Sigma) at room temperature for 2 h. The DAB-peroxidase reaction (0.05% in 0.01 M PBS, pH 7.4, Sigma) was carried out for 2–8 min and mounted onto gelatin-coated slides, dried, dehydrated, cleared with xylene, and covered with neutral balsam.

To follow up on the fate of the EGFP-labeled transplanted stem cells, immunofluorescent detection was performed. NeuN was used as a mature neuronal marker, Darpp32 as the medium-sized striatal projection neurons marker, GFAP as the mature astrocyte marker, and Iba-1 as the microglia marker. Sections were blocked and incubated overnight at 4°C with primary antibodies as mentioned above. After washing in PBS, sections were incubated with fluorescence-conjugated secondary antibodies (Alexa Fluor 594, 1∶500; Invitrogen) for 2 h at room temperature. Sections were washed and counterstained with the nuclear dye hoechst33258 (1∶1000, Sigma) for 15 min. Fluorescence-labeled sections were viewed and images captured with a confocal microscope (Olympus).

### 8. Western blot

Western blotting was carried out for the marker proteins for each cell type examined. Rats were killed by decapitation after being anesthetized, and the striatum was extracted and homogenized in a lysis buffer to which protease inhibitors had been freshly added. The homogenate was centrifuged at 1500 g for 25 min, and protein concentration was determined by using a Bio-Rad DC protein assay (Bio-Rad, Hercules, CA). Samples were separated by sodium dodecyl sulfate-polyacrylamide gel electrophoresis (1% SDS-PAGE) and transferred to PVDF membranes (Millipore). Membranes were incubated in blocking buffer (5% skim milk in TBST), then with mouse anti- NeuN (1∶2000, Millipore), rabbit anti- Darpp32 (1∶1000, Millipore), mouse anti-GFAP (1∶2000, Santa Cruz Biotechnology), and rabbit anti- Iba-1 (1∶2000, Millipore), or rabbit anti -β-actin (1∶2000, Millipore) in TBST overnight at 48°C. Incubated membranes were then treated with secondary antibody conjugated with horseradish peroxidase in TBST for 2 hr at 37°C. Blots were developed by enhanced chemiluminescence and digitally scanned. The optical density of each resulting labeled band was measured in an image analysis program (Image pro-Plus 6.0).

### 9. Data collection and statistical analysis

Histologic analysis of rat-brain volumes was performed as previously described [27]. Continuous 50 µm coronal brain sections were taken from levels corresponding approximately to the interaural plane from 10.70 to 8.74 mm [according to the atlas of [28]] and were used for volumetric analysis. The areas of the striatum as determined by Nissl staining were calculated from each continuous section and total volumes were measured by integrating each section area and depth using Image-Pro Plus 6.0 software.

For immunohistochemistry analysis, eight sections for each rat were analyzed per neuron type. Quantitative analysis of the number of positively stained cells with NeuN, Darpp32, GFAP and Iba-1 was performed on adjacent coronal sections of the striatum. For NeuN and Darpp32, the number of labeled perikarya was counted in six randomly selected areas (0.01 mm^2^ for each) in striatum for each section. For GFAP and Iba-1, the integral optical density (IOD) of positive cells in six randomly selected areas (0.01 mm^2^ for each) in striatum was measured with an image analysis program (Image pro-Plus 6.0).

Comparisons were performed using one-way ANOVA and unpaired t-test. Data are presented as mean±SD, and differences considered significant at *p<*0.05.

## Results

### 1. Transplantation of iPSC improves recovery of learning and memory deficits induced by QA

**Figure 1:**
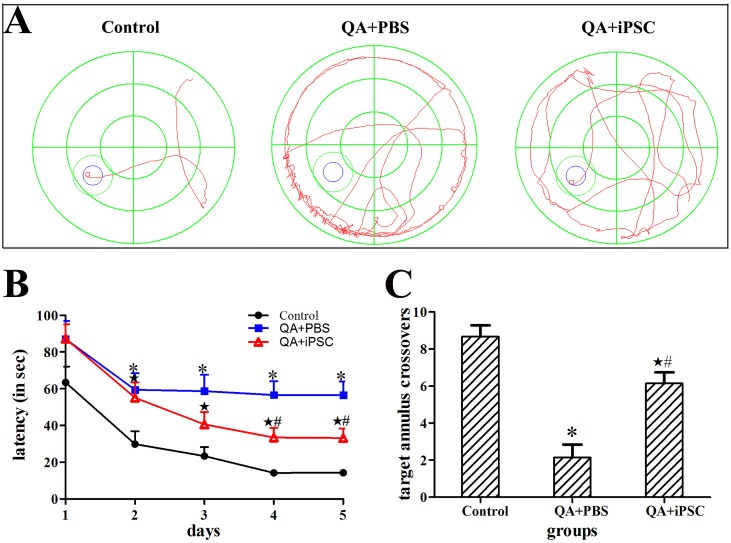
Transplanted iPSC improved functional recovery by Morris water maze testing. (A) The swim tracks of the rats during Morris water maze testing. The QA+iPSC rat obviously spent less time searching for the platform than the QA+PBS rat. (B) Comparison of the latency to find the platform among the three groups in Morris water maze testing. (C) Comparison of the crossovers among the three groups in probe test of Morris water maze testing. The 3 groups are presented as control (saline injection), QA+PBS (QA injection with PBS transplantation); and QA+iPSC (QA injection with iPSC transplantation) groups. Error bars represent SD, and * *P<*0.05, compared between control and QA+PBS groups; ★ *P<*0.05, compared between control and QA+iPSC groups; # *P<*0.05, compared between QA+PBS and QA+iPSC groups.

In the Morris water maze task, the QA treated rats moved more along the wall of the pool and were significantly slower than controls in locating the hidden platform. However, in the iPSC-transplanted group, the animals could find the platform more quickly than the QA-treated rats (Fig. 1A). On the first day of testing, most of the animals in these three groups could not find the platform. After the initial trial, QA-treated rats showed consistently longer latencies in locating the hidden platform during the 5 days of testing, but, compared with the first day of testing, the iPSC-transplanted animals showed to find the platform sooner on Day 2 to Day 5. Analyses of the escape latency for hidden platform trials showed a significant difference among the three groups (compared between control and QA-treated groups on Day 2 to Day 5, *p<*0.05; compared between QA-treated and iPSC-transplanted groups on Day 4 to Day 5, *p<*0.05; compared between control and iPSC-transplanted groups on Day 2 to Day 5, *p<*0.05, Fig. 1B, Table S1A in File S1).

On the following probe test without the platform, the control rats spent more time in the target quadrant, and the number of the animals crossing the target annulus was nearly 8; whereas the QA treated rats swam mainly in the periphery of the pool, and the mean crossover was only 2. In the iPSC-transplanted group, the mean crossovers could be up to 6 times. Statistical analysis showed a significant difference of the crossovers among the three groups (compared between the control and QA-treated groups, *p<*0.001; compared between QA-treated and iPSC-transplanted groups, *p<*0.001; compared between the control and iPSC-transplanted groups, *p<*0.05, Fig. 1C, Table S1B in File S1).

### 2. iPSC transplantation results in enhanced glucose metabolism of the lesioned striatum

**Figure 2:**
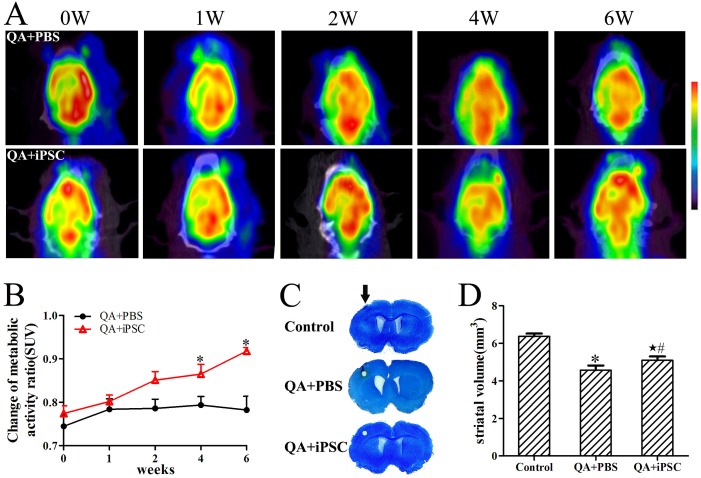
Enhanced glucose metabolism and decreased striatal atrophy following iPSC transplantation. (A) Serial PET images demonstrating metabolism recovery after stem cell treatment for QA-treated rats. Images are shown in axial view. Scale was set according to signal intensity. (B) Semiquantitative analysis of variance of glucose metabolism after stem cell transplantation in each group. (C) Photomicrographs show the difference in striatal volumes in unilateral lesion rat. There was no striatal atrophy in the control group, but the striatal volumes in the lesion side of QA+PBS and QA+iPSC groups were significantly decreased compared with the contralateral side. Arrow indicates surgery sites in each group. (D) Quantification of striatal volumes show increased volumes in the QA+iPSC group. The 3 groups are presented as control (saline injection), QA+PBS (QA injection with PBS transplantation); and QA+iPSC (QA injection with iPSC transplantation) groups. Error bars represent SD, and * *P<*0.05, compared between control and QA+PBS groups; ★ *P<*0.05, compared between control and QA+iPSC groups; # *P<*0.05, compared between QA+PBS and QA+iPSC groups.

To document that the iPSC implant corresponds to enhanced glucose metabolic activity, ^18^F-FDG small-animal PET/CT scans were performed sequentially at 0, 1, 2, 4 and 6 weeks after iPSC transplantation. The ^18^F-FDG PET/CT scans allowed the visualization and quantification of glucose metabolism throughout the brain at each time point (Fig. 2). For the animals included in this study, the lesion-to-normal homologous contralateral radioactivity ratio was used for semiquantitative analysis. In rats that were injected with QA into the left striatum and imaged 10 days later, there was a marked asymmetry of FDG uptake in the striatum, compared to the non-lesioned right hemisphere (Fig. 2A). From 1 week to 6 weeks after transplantation, glucose metabolism in the striatum of QA-treated animals remained unchanged. In contrast, the glucose metabolism in iPSC-transplanted rats showed a slight increase, resulting in a growth of the radioactivity ratio (Figs. 2A, 2B). This growth persisted through the four-week scan, suggesting that the transplant protected the ipsilateral striatum from metabolic decline. Analysis of the radioactivity ratios demonstrated no significant differences between the QA-treated and iPSC-transplanted groups at either 1 or 2 weeks after transplantation (*p>*0.05, Fig. 2B). However, the radioactivity ratios were significantly increased in the iPSC-transplanted group at both 4 and 6 weeks after stem cell transplantation (*p<*0.001, Fig. 2B, Table S2 in File S1), indicating that transplantation of iPSC increased glucose metabolism in the lesioned area.

In addition, there was no focal abnormal increase in glucose metabolism in the cerebral lesioned area, thus indicating no tumor or teratoma formation at 6 weeks after stem cell transplantation.

### 3. iPSC transplantation improves striatum volume after QA-induced excitotoxicity

At 10th day after injection of QA, the striatal atrophy and lateral veniricle dilation were severe in the QA-injection side compared with the contra-lateral side. Nissl staining showed that a clear lesion was located in the striatum and neuronal loss was observed in the lesion area. Striatum volumes were calculated by Nissl staining to evaluate the effects of iPSC transplantation. Six weeks after surgery, striatum volumes were not changed in the control group, but were obviously decreased in the QA-lesioned and iPSC-transplanted groups (Fig. 2C). However, striatum volumes in iPSC-transplanted group were partially recovered in comparison with QA-lesioned group (Fig. 2D). The striatum volume of the control group was approximately 6.37±0.15 mm^3^, while in the QA-lesioned and iPSC -transplanted groups striatum volume was 4.57±0.25 mm^3^ and 5.10±0.20 mm^3^, respectively. The statistical analysis showed a significant difference among these three groups (*P<*0.001 for control and QA groups, *P<*0.05 for QA and iPSC-transplanted groups, *P<*0.05 for control and iPSC-transplanted groups, Fig. 2D, Table S3 in File S1).

### 4. Histological confirmation of neural protection after iPSC transplantation

#### 4.1 Neural loss and gliosis induced by QA injection

**Figure 3:**
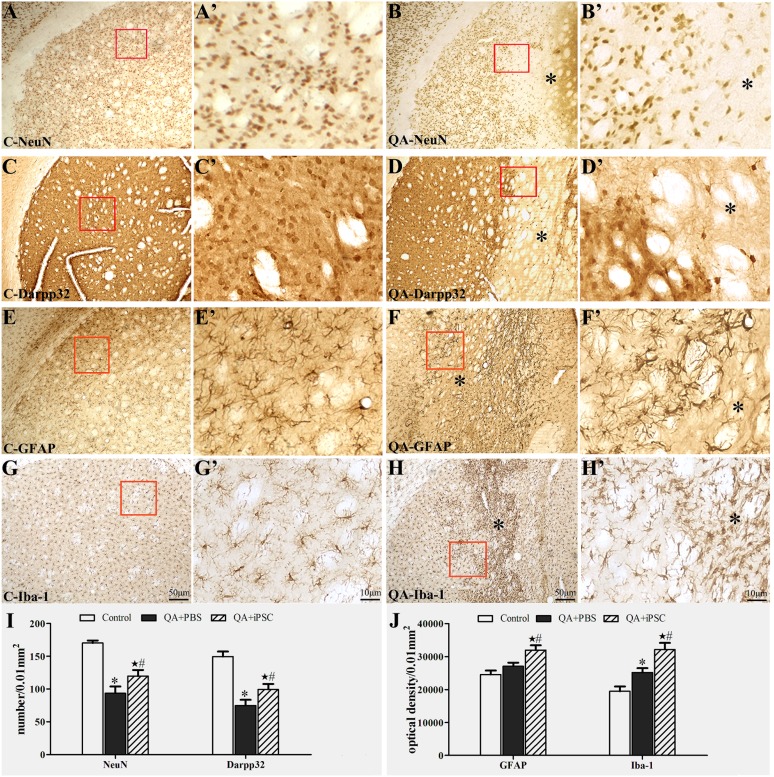
Neuronal loss and gliosis in striatum induced by QA injection. (A, C, E, G and A', C', E', G') Immunohistochemical staining for NeuN (A–A'), Darpp32 (B–B'), GFAP (C–C'), Iba-1 (D–D') in striatum in the control rats. (B, D, F, H and B', D', F', H') Immunohistochemical staining for NeuN (B–B'), Darpp32 (D–D'), GFAP (F–F'), Iba-1 (H–H') in striatum in the QA-treated rats, which showed the loss of NeuN- and Darpp32-positive neurons and proliferations of astrocyte and microglia in the lesion area. A–H were the same magnification; A'–H' were the same magnification; A'–H' were the higher magnification views of the red box in A–H. (I) Comparison of the number of NeuN- and Darpp32-positive neurons in striatum among the three groups. (J) Comparison of the optical density of GFAP- and Iba-1-positive glial cells in striatum among the three groups. The 3 groups are presented as control (saline injection), QA+PBS (QA injection with PBS transplantation); and QA+iPSC (QA injection with iPSC transplantation) groups. Error bars represent SD, and * *P<*0.05, compared between control and QA+PBS groups; ★ *P<*0.05, compared between control and QA+iPSC groups; # *P<*0.05, compared between QA+PBS and QA+iPSC groups.

To evaluate QA-induced striatal lesion, immunohistochemical (NeuN, Darpp32, GFAP and Iba-1) methods were used. NeuN was used as a mature neuronal marker, and Darpp32 as the medium-sized striatal projection neurons marker. In QA-treated rats, a clear lesion area was obviously located in the striatum. NeuN and Darpp32 immunolabeling confirmed a serious loss of neurons in the lesion core (Fig. 3B, 3D). Quantitative data showed that the number of striatal neurons in the QA group was 93.5±10.4/0.01 mm^2^ (NeuN) and 74.9±8.6/0.01 mm^2^ (Darpp32), respectively, corresponding to 170.2±3.9/0.01 mm^2^ (NeuN) and 149.5±7.8/0.01 mm^2^ (Darpp32) in the control. The statistical analysis showed a significant difference between the two groups (*P<*0.001 for NeuN, *P<*0.001 for Darpp32, Fig. 3I, Table S4 in File S1). Furthermore, histological detection in astrocyte and microglia were carried out using GFAP and Iba-1 immunohistochemistry. In the control group, astrocyte and microglia were scattered in striatum uniformly. After QA treatment, there were numerous glial cell proliferations in the lesion core (Fig. 3F, 3H). Statistical analysis showed that in the QA group, the optical density of GFAP and Iba-1 in striatum was 27045±1093/0.01 mm^2^ and 25137±1359/0.01 mm^2^, respectively, which showed a significant difference compared with the controls for Iba-1 (19456±1453/0.01 mm^2^, *P<*0.05) but not for GFAP (24560±1203/0.01 mm^2^, *P>*0.05, Fig. 3J, Table S5 in File S1).

#### 4.2 Transplanted cells survive and differentiate into neurons and astrocytes in lesioned striatum

**Figure 4:**
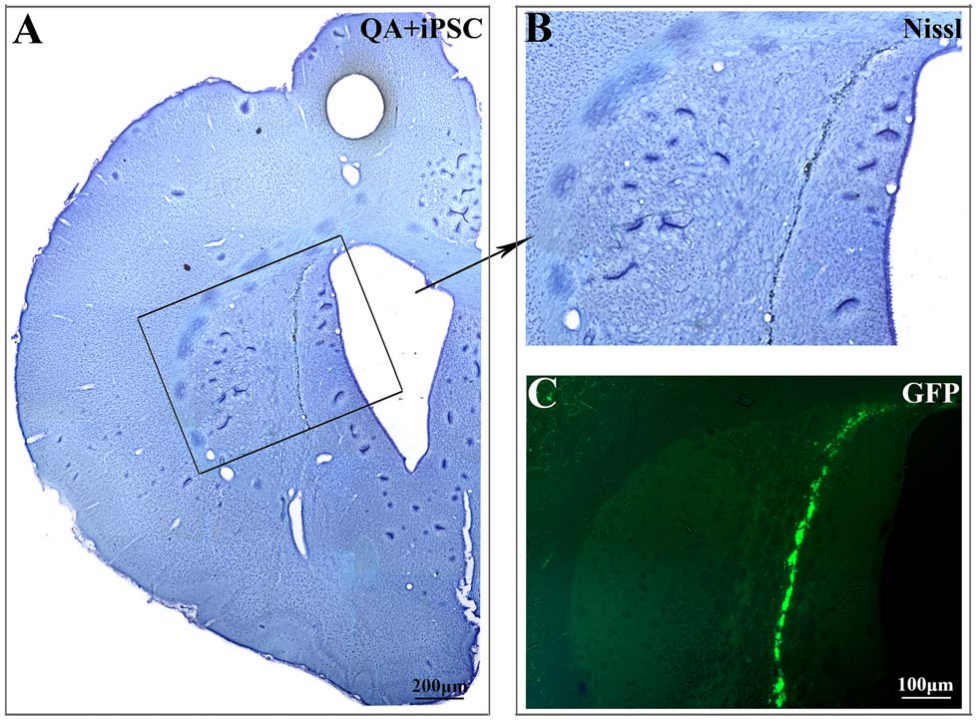
Transplanted iPSC migrated into the lesioned striatum after QA injection. (A, B) Nissl staining of striatum after iPSC transplantation. B was the higher magnification views of the black box in A. (C) Migration and distribution of the transplanted iPSC in QA-lesioned striatum were observed under fluorescence microscope. B and C were the same magnification.

We next investigated the distribution and differentiation of the transplanted iPSC in QA-lesioned striatum. As labeled by EGFP, transplanted iPSC could be identified under fluorescence microscope. A number of transplanted iPSC appeared to have migrated from lateral ventricle to the lesioned striatum, and the cells spread out into the lesioned area (Fig. 4). While, there is no EGFP-marked cell can be detected in contra-lateral side.

**Figure 5:**
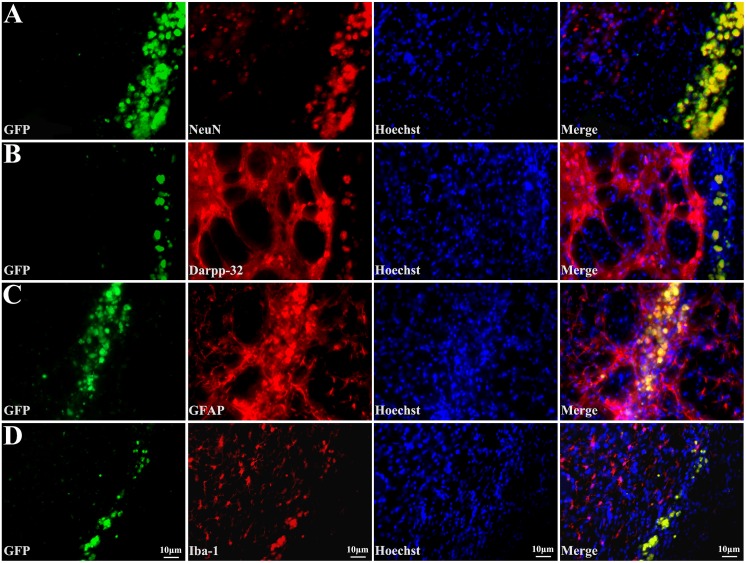
Transplanted cells differentiate into neurons and astrocytes in lesioned striatum. Transplanted iPSCs show green fluorescence; immunostaining with antibodies against NeuN (A), Darpp32 (B), GFAP (C) and Iba-1 (D) show red fluorescence; nuclei stained with Hoechst 33258 show blue fluorescence; and merged images show that engrafted iPSC express neuron, projection neuron, astrocyte, or microglia features.

Furthermore, we determined the cell types of differentiated iPSC surviving in the QA impaired striatum. Confocal microscope images showed a larger number of GFP-labeled cells in the QA-lesioned striatum co-expressing NeuN (Fig. 5A), Darpp32 (Fig. 5B), GFAP (Fig. 5C) and Iba-1 (Fig. 5D). Then, we counted the number of NeuN- and Darpp32-expressing cell in striatum. In the iPSC-transplanted animals, the number of NeuN-positive cells was significantly higher than that in the QA-injected group (119.8±9.3/0.01 mm^2^ vs 93.5±10.4/0.01 mm^2^; *P<*0.05, Fig. 3I). The number of Darpp32-expressing cells in the iPSC-transplanted group was more than that in the QA-treated group (99.4±8.3/0.01 mm^2^ vs 74.9±8.6/0.01 mm^2^) and there was statistically significant difference between the two groups (*P<*0.05, Fig. 3I, Fig. 3J). The IOD of GFAP and Iba-1 in striatum was also quantified. Expression of GFAP in the iPSC-transplanted striatum was significantly higher than that in the QA-treated group (31934±1493/0.01 mm^2^ vs 27045±1093/0.01 mm^2^; *P<*0.05). Meanwhile, the IOD of Iba-1 in the iPSC-transplanted group was significantly higher than that of the QA-injected group (332143±2019/0.01 mm^2^ vs 25137±1359/0.01 mm^2^; *P<*0.001, Fig. 3J). Analyses of the four cell types between the control and iPSC-transplanted groups also showed a significant difference (*P<*0.05 for NeuN and Darpp32, *P<*0.001 for GFAP and Iba-1, Fig. 3I, Fig. 3J, Table S4 and S5 in File S1).

### 5. Changes of protein marker in striatal neuron and gliocyte after iPSC transplantation

**Figure 6:**
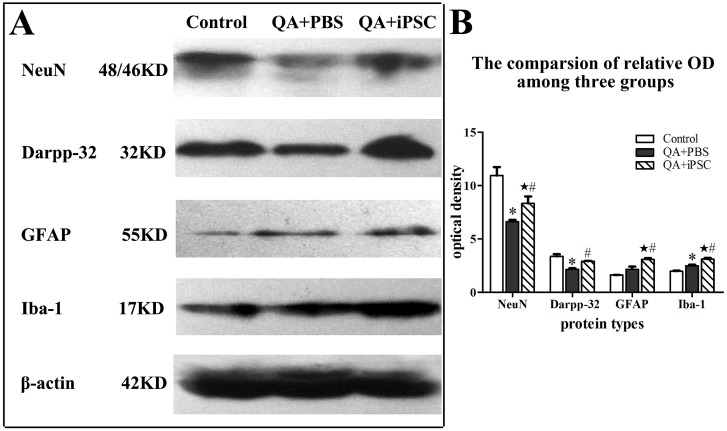
Increased protein expression of neurons and glia cell in striatum after iPSC transplantation. (B) was the semiquantitative analysis of (A) expressed as relative optical density, which showed that the levels of NeuN, Darpp32, GFAP and Iba-1 proteins increased after iPSC transplantation. The 3 groups are presented as control (saline injection), QA+PBS (QA injection with PBS transplantation); and QA+iPSC (QA injection with iPSC transplantation) groups. Error bars represent SD, and *P<0.05, compared between control and QA+PBS groups; ★ *P<*0.05, compared between control and QA+iPSC groups; # *P<*0.05, compared between QA+PBS and QA+iPSC groups.

We then examined the protein expression of NeuN, Darpp32, GFAP and Iba-1 in striatum after iPSC transplanted for six weeks. Western blotting revealed that treated with QA significantly decreased NeuN and Darpp32 protein in rat striatum, but iPSC-transplanted rat had higher levels of these molecules than the QA-treated rat (*p<*0.05; Fig. 6). QA-treated rat expressed increased levels of both GFAP and Iba-1 in striatum compared with the control animals, and iPSC transplantation could enhance their expression more significantly than the QA-treated rats (*p<*0.05; Fig. 6), indicating that iPSC activated the proliferation of gliocyte. Statistical analysis also showed a significant difference between the control and iPSC-transplanted groups for NeuN, GFAP and Iba-1, but not Darpp32 (*p<*0.05 for NeuN; *p<*0.001 for GFAP and Iba-1; *p>*0.05 for Darpp32, Fig. 6, Table S6 in File S1).

## Discussion

In the present study, we found that transplantation of iPSCs reduced learning and memory dysfunction in QA-lesioned rat, as determined by Morris water maze. Within the damaged striatum, a large number of iPSCs could migrate into the damaged striatal region and underwent differentiation, as shown by the expression markers for mature neurons, striatal medium spiny projection neurons, astrocytes and microglia. Moreover, the potential therapeutic effect of iPSCs was evaluated by serial ^18^F-FDG PET/CT scans.

Elevated cerebral glucose uptake reflects a higher synaptic activity in the brain if no inflammatory or malignant process is present [29]. Reduction of the cerebral glucose metabolism, observed by PET imaging, is confirmed a well-known feature in symptomatic HD and the preclinical gene carrier state [30]. Therefore, monitoring glucose utilization in animal models of HD, and also in patients, can provide useful information about neuronal functional deficit before and after therapeutic interventions. It has reported that cystamine-induced neuroprotection in R6/2 transgenic mice can be monitored by micro PET-[^18^F] FDG in the striatum, cortex and cerebellum [31]. Here, we used micro ^18^F-FDG PET imaging to investigate whether iPSC transplantation can ameliorate the cerebral energy metabolism in the striatum of rat model of HD. The similar study has been verified in the cerebral ischemia. Wang et al reported that serial ^18^F-FDG small-animal PET demonstrated metabolic recovery after iPSC and ESC transplantation in a rat model of cerebral ischemia [12]. Clinically, a previous study demonstrated enhanced glucose metabolism in some patients that had undergone transplantation of fetal striatum. Although the sample size was small, the metabolic rates, observed by PET appeared to correlate with improved clinical status [7]. In the present study, by using a rat model of Huntington's disease, we were able to find increased glucose metabolic activity in the striatum-lesioned area during the 6-wk period after iPSC transplantation under serial ^18^F-FDG PET scans. Thus, PET seems likely to be one of the best-suited modalities to evaluate stem cell therapy, and it can be used in patients clinically for both cells trafficking and monitoring the response to therapy.

In the injured brain, some upregulated environmental elements may account for the migration of endogenous neural stem cells or transplanted immortalized, neonate-derived neural precursor cells to the lesioned region and their differentiation into neurons [32]. In previous studies, brain injury induced neurogenesis and enhanced neuronal migration to the lesioned region to enhance proliferation of correct cell types, such as Dcx-expressing neuroblasts, to reconstruct the damaged cell architecture, as seen after stroke [33] and HD [34]. In the present study, immunohistochemical analyses indicated that many transplanted cells survived and integrated close to the injured striatum and that most of the cells expressed protein markers for parenchymal cells such as neurons and astrocytes. What are the mechanisms or factors that promote reduced deficits with iPSC transplantation after HD? One possibility is that the transplanted stem cells integrate into the tissue, replace damaged cells, and reconstruct the neural circuitry. Previous reports demonstrated that human induced PSC-derived dopaminergic neurons have been shown to survive and improve behavioural impairments after intrastriatal transplantation in an animal model of Parkinson's disease [16]. Another reasonable explanation is that changes in the microenvironment may contribute to tissue protection and repair [35] or that the interaction of grafted stem cells with the host brain may lead to production of trophic factors [36]. For example, increased neurogenesis and neuroprotection by neurotrophic or growth factors, and new synapse formation with reorganization, have been suggested by stroke models [37]. The decreased striatal atrophy, the increased glucose metabolic activity and the improved functional recovery of the HD model in our study also suggest that iPSC transplantation protects the host brain from further destruction.

Recently, the role of glial cells in integrity of the central nervous system had been well elucidated, and accumulating evidence suggested that glial cells were critical to neuronal survival [38], [39]. Gliocyte populations in different brain regions have been shown to have important roles in neurogenic support [40]. A recent report revealed a surprising outcome where SVZ-mediated astrogenesis may be beneficial over neurogenesis in a period after cortical injury, in agreement with potentially protective roles for astrocytes during recovery process in the spinal cord [41]. In both cultured astrocytes and HD mouse brains, mutant huntingtin reduces glial glutamate uptake and glial cells protected neurons against mutant htt-mediated neurotoxicity [42], suggesting that dysfunction of glial cell may critically contribute to neuronal excitotoxicity in HD. In addition, in injured striatum, mutant huntingtin may affect the production of chemokines and neurotrophic factors such as tumor necrosis factor alpha (TNF-a) [43], glial-derived neurotrophic factor (GDNF) [44] and nerve growth factor (NGF) [45] from glial cells. In our present study, a large number of iPSCs that differentiated into astrocyte and microglia may contribute to chemokine secretion and iPSCs transplantation may provide a beneficial environment that attracts glial cell activation and proliferation. Once the numbers of activated glial cells are boosted, their trophic support will slow the excitotoxic damage to GABAergic neurons in the striatum.

## Conclusion

In summary, we have demonstrated that iPSCs survive, migrate into injured striatum and differentiate into neurons and glial cells after transplantation in the QA-induced HD model, accompany with the decreased striatal atrophy, the increased glucose metabolic activity and the improved functional recovery. Furthermore, we have demonstrated that^18^F-FDG PET scan seems likely to be a feasible modality to evaluate stem cell therapy in HD. Collectively, this study demonstrates the therapeutic potential of iPSCs for cell replacement therapy and indicates that iPSCs may provide an alternative cell source for transplantation therapy in the treatment of HD and other neurodegenerative diseases.

## Supporting Information

File S1 **This file contains Tables S1–S6.** Table S1–S6 showed the raw data of this study. Table S1 showed the raw data of Morris water maze testing, A is the latency and B is the target annulus crossovers. Table S2 showed the raw data of SUV of PET/CT. Table S3 showed the raw data of the striatal volume (mm^3^). Table S4 showed the raw data of the number of NeuN and Darpp32/0.01 mm^2^ in striatum. Table S5 showed the raw data of the optical density of GFAP and Iba-1/0.01 mm^2^ in striatum. Table S6 showed the raw data of the optical density of Western blot.
(DOCX)
