# Supplementary material for: Transplantation of Induced Pluripotent Stem Cells Improves Functional Recovery in Huntington's Disease Rat Model
Source: PLoS One. 2014 Jul 23;9(7):e101185. doi: 10.1371/journal.pone.0101185 (PMC4108311; doi:10.1371/journal.pone.0101185)
Supplement: File S1 — This file contains Tables S1–S6. Table S1–S6 showed the raw data of this study. Table S1 showed the raw data of Morris water maze testing, A is the latency and B is the target annulus crossovers. Table S2 showed the raw data of SUV of PET/CT. Table S3 showed the raw data of the striatal volume (mm3). Table S4 showed the raw data of the number of NeuN and Darpp32/0.01 mm2 in striatum. Table S5 showed the raw data of the optical density of GFAP and Iba-1/0.01 mm2 in striatum. Table S6 showed the raw data of the optical density of Western blot. (DOCX) [file pone.0101185.s001.docx]

**Raw data of “Transplantation of induced pluripotent stem cells improves functional recovery in Huntington's disease rat model”**

**Table S1: Raw data of Morris water maze.**

**A, Latency (in sec)**

| days | 1 | 2 | 3 | 4 | 5 |
| --- | --- | --- | --- | --- | --- |
| Control | 119.875 | 12.028 | 13.777 | 12.87 | 3.4578 |
|  | 90.348 | 8.86 | 8.205 | 18.13 | 4.6002 |
|  | 41.893 | 11.956 | 8.747 | 11.53 | 6.5688 |
|  | 7.694 | 6.998 | 18.64 | 30.24 | 7.1706 |
|  | 6.797 | 11.045 | 42.337 | 3.39 | 7.2522 |
|  | 98.105 | 5.165 | 10.178 | 17.84 | 9.9144 |
|  | 5.266 | 10.17 | 10.294 | 9.72 | 11.3526 |
|  | 20.021 | 3.833 | 9.299 | 14.11 | 11.7606 |
|  | 119.875 | 34.37 | 15.009 | 7.03 | 11.7912 |
|  | 40.47 | 23.084 | 28.435 | 13.79 | 11.8116 |
|  | 30.338 | 12.468 | 68.908 | 14.78 | 13.056 |
|  | 119.878 | 7.55 | 48.724 | 16.18 | 13.1274 |
|  | 119.655 | 24.527 | 8.309 | 18.89 | 13.1682 |
|  | 82.69 | 9.623 | 10.745 | 7.11 | 14.0658 |
|  | 29.42 | 14.216 | 12.692 | 4.51 | 14.39796 |
|  | 119.876 | 15.663 | 11.185 | 27.84 | 15.08163 |
|  | 50.464 | 58.789 | 7.104 | 20.75 | 14.67 |
|  | 19.8 | 6.05 | 3.948 | 6.44 | 17.20408 |
|  | 2.986 | 13.562 | 25.172 | 11.13 | 18.09184 |
|  | 119.801 | 6.133 | 11.384 | 11.58 | 18.20408 |
|  | 83.054 | 119.492 | 15.575 | 19.38 | 18.5 |
|  | 57.317 | 97.236 | 13.897 | 12.91 | 19.27551 |
|  | 14.22 | 13.127 | 72.099 | 16.86 | 19.77551 |
|  | 119.829 | 119.875 | 17.062 | 17.73 | 21.17347 |
|  | 119.873 | 118.701 | 107.113 | 12.8 | 24.6 |
|  | 53.924 | 12.031 | 9.191 | 11.56 | 30.85714 |
|  | 20.029 | 48.238 |  |  |  |
|  |  | 11.069 |  |  |  |
| QA+PBS | 119.791 | 119.871 | 119.875 | 92.62 | 8.428572 |
|  | 119.879 | 119.789 | 119.872 | 119.61 | 14.67 |
|  | 119.877 | 119.809 | 119.888 | 97.16 | 11.45918 |
|  | 119.878 | 119.875 | 119.874 | 119.46 | 13.16327 |
|  | 119.411 | 119.876 | 36.005 | 57.17 | 17.27551 |
|  | 119.802 | 54.138 | 40.14 | 117.48 | 20.82653 |
|  | 119.827 | 29.244 | 29.426 | 102.09 | 32.27551 |
|  | 7.993 | 49.875 | 23.755 | 37.04 | 37.79592 |
|  | 119.879 | 51.644 | 119.871 | 72.44 | 41.45 |
|  | 115.562 | 7.47 | 119.879 | 73.52 | 51.97959 |
|  | 119.883 | 104.675 | 119.878 | 48.28 | 52.12 |
|  | 119.883 | 119.876 | 26.594 | 16.93 | 59.62626 |
|  | 119.828 | 10.397 | 17.065 | 12.9 | 62.47475 |
|  | 119.805 | 31.14 | 14.339 | 72.44 | 73.17171 |
|  | 112.873 | 47.474 | 15.789 | 73.52 | 73.17171 |
|  | 15.747 | 5.709 | 4.918 | 20.41 | 74.26263 |
|  | 119.889 | 61.142 | 108.502 | 5.52 | 74.26263 |
|  | 65.766 | 41.443 | 35.218 | 50.94 | 89.56 |
|  | 119.769 | 107.528 | 24.3 | 61.85 | 94.10101 |
|  | 119.827 | 28.711 | 7.981 | 11.01 | 98.14141 |
|  | 11.6 | 42.597 | 94.505 | 11.23 | 98.09 |
|  | 3.183 | 17.228 | 57.855 | 8.26 | 118.6667 |
|  | 30.956 | 13.253 | 72.976 | 5.52 | 120.6667 |
|  | 11.513 | 4.495 | 18.489 | 59.03 | 120.8182 |
|  | 6.013 |  | 50.665 | 31.63 | 5.632653 |
|  |  |  | 9.839 | 93.16 | 5.632653 |
|  |  |  |  |  |  |
|  |  |  |  |  |  |
| QA+iPSC | 33.907 | 10.621 | 15.421 | 13.9 | 7.515152 |
|  | 97.232 | 23.95 | 21.562 | 37.04 | 13.0303 |
|  | 67.633 | 12.682 | 46.599 | 19.11 | 14.0404 |
|  | 9.544 | 9.297 | 6.473 | 6.44 | 14.0404 |
|  | 119.798 | 119.874 | 57.421 | 53.09 | 15.25253 |
|  | 119.871 | 72.626 | 48.674 | 29.85 | 16.32323 |
|  | 56.34 | 88.079 | 52.733 | 50.81 | 17.10101 |
|  | 119.848 | 6.48 | 7.561 | 57.17 | 19.30303 |
|  | 119.881 | 32.48 | 11.264 | 13.9 | 20.61616 |
|  | 89.69 | 18.388 | 16.298 | 37.04 | 21.4929 |
|  | 63.466 | 20.381 | 11.047 | 119.92 | 27.5616 |
|  | 118.894 | 42.324 | 12.387 | 16.16 | 27.7695 |
|  | 113.204 | 119.814 | 63.105 | 7.44 | 29.5515 |
|  | 108.608 | 119.875 | 119.814 | 21.71 | 36.6696 |
|  | 14.232 | 15.97 | 12.365 | 48.28 | 36.6696 |
|  | 109.244 | 10.516 | 5.357 | 16.93 | 47.7972 |
|  | 22.391 | 119.877 | 14.336 | 12.9 | 50.3019 |
|  | 119.878 | 28.196 | 20.89 | 15.1 | 50.4306 |
|  | 15.226 | 105 | 17.505 | 7.37 | 52.5591 |
|  | 119.881 | 81.047 | 18.375 | 64.8 | 56.5983 |
|  | 113.555 | 17.063 | 31.277 | 2.87 | 61.2315 |
|  | 119.877 | 23.663 | 83.783 | 20.41 | 64.152 |
|  | 119.812 | 37.105 | 42.875 | 5.52 | 89.0406 |
|  | 119.875 | 21.547 | 15.091 | 28.05 | 118.7208 |
|  | 119.874 | 119.791 | 119.877 | 50.94 | 2.89899 |
|  | 95.718 | 119.144 | 105.332 | 61.85 | 5.575758 |
|  | 30.198 | 34.891 | 90.125 | 89.94 | 6.505051 |
|  |  | 112.226 | 70.688 | 27.84 | 7.444445 |

**B, target annulus crossovers**

| Control |  | 9 | 6 | 10 | 10 | 8 | 9 |
| --- | --- | --- | --- | --- | --- | --- | --- |
| QA+PBS | 0 | 1 | 0 | 4 | 4 | 2 | 4 |
| QA+iPSC | 6 | 8 | 5 | 8 | 5 | 7 | 4 |

**Table S2: Raw data of SUV of PET/CT**

| weeks | 0 | 1 | 2 | 4 | 6 |
| --- | --- | --- | --- | --- | --- |
| QA+PBS | 0.6842105 | 0.7826087 | 0.7368421 | 0.76 | 0.84 |
|  | 0.8235294 | 0.8333333 | 0.7222222 | 0.8 | 0.8846154 |
|  | 0.6842105 | 0.8333333 | 0.883 | 0.875 | 0.84 |
|  | 0.8333333 | 0.65 | 0.76 | 0.72 | 0.65 |
|  | 0.8 | 0.75 | 0.8 | 0.8461539 | 0.7142857 |
|  | 0.8421053 | 0.7 | 0.875 | 0.8461539 | 0.65 |
|  | 0.6666667 | 0.8571429 | 0.7058824 | 0.7058824 | 0.8214286 |
|  | 0.6818182 | 0.8571429 | 0.7647059 | 0.7647059 | 0.75 |
|  | 0.6842105 | 0.7894737 | 0.8235294 | 0.8235294 | 0.8888889 |
| QA+iPSC | 0.7272727 | 0.7941176 | 0.85 | 0.8 | 0.8888889 |
|  | 0.6818182 | 0.7941176 | 0.75 | 0.79 | 0.8823529 |
|  | 0.7727273 | 0.8529412 | 0.8241379 | 0.8 | 0.9444444 |
|  | 0.7586207 | 0.75 | 0.82 | 0.8260869 | 0.9375 |
|  | 0.7931035 | 0.7368421 | 0.92 | 0.9565217 | 0.9375 |
|  | 0.8214286 | 0.7894737 | 0.79 | 0.9130435 | 0.8823529 |
|  | 0.7368421 | 0.8 | 0.9130435 | 0.9244444 | 0.9285714 |
|  | 0.8411765 | 0.8333333 | 0.9130435 | 0.9411765 | 0.9285714 |
|  | 0.8333333 | 0.8666667 | 0.8765218 | 0.8333333 | 0.9285714 |

**Table S3: Raw data of the striatal volume (mm^3^)**

| Control | QA+PBS | QA+iPSC |
| --- | --- | --- |
| 6.5 | 4.74 | 5.36 |
| 6.16 | 4.82 | 4.99 |
| 6.4 | 4.36 | 5.14 |
| 6.42 | 4.35 | 4.89 |

**Table S4: Raw data of the number of NeuN and Darpp32 / 0.01mm^2^ in striatum**

|  | control | QA+PBS | QA+Ipsc |  | control | QA+PBS | QA+Ipsc |
| --- | --- | --- | --- | --- | --- | --- | --- |
| NeuN | 175 | 110 | 129 | Darpp32 | 139 | 62 | 99 |
|  | 173 | 88 | 125 |  | 158 | 81 | 92 |
|  | 167 | 79 | 118 |  | 160 | 81 | 95 |
|  | 166 | 96 | 125 |  | 143 | 73 | 98 |
|  | 171 | 97 | 138 |  | 139 | 63 | 110 |
|  | 168 | 86 | 128 |  | 138 | 79 | 116 |
|  | 179 | 97 | 123 |  | 149 | 71 | 106 |
|  | 167 | 108 | 110 |  | 161 | 70 | 89 |
|  | 173 | 81 | 124 |  | 153 | 77 | 87 |
|  | 171 | 75 | 118 |  | 152 | 60 | 100 |
|  | 174 | 92 | 114 |  | 146 | 81 | 95 |
|  | 165 | 90 | 126 |  | 148 | 74 | 98 |
|  | 173 | 96 | 128 |  | 149 | 78 | 111 |
|  | 172 | 101 | 104 |  | 151 | 63 | 109 |
|  | 161 | 104 | 109 |  | 142 | 82 | 106 |
|  | 168 | 112 | 125 |  | 159 | 64 | 89 |
|  | 170 | 91 | 115 |  | 144 | 83 | 93 |
|  | 169 | 75 | 116 |  | 160 | 89 | 96 |
|  | 175 | 95 | 119 |  | 160 | 76 | 107 |
|  | 175 | 118 | 105 |  | 157 | 79 | 106 |
|  | 169 | 89 | 106 |  | 139 | 83 | 98 |
|  | 176 | 95 | 107 |  | 148 | 84 | 107 |
|  | 173 | 88 | 106 |  | 145 | 82 | 87 |
|  | 170 | 92 | 120 |  | 149 | 68 | 91 |
|  | 169 | 79 | 115 |  | 147 | 76 | 98 |
|  | 166 | 89 | 121 |  | 151 | 90 | 85 |
|  | 169 | 106 | 139 |  | 157 | 82 | 94 |
|  | 163 | 93 | 136 |  | 158 | 69 | 103 |
|  | 172 | 89 | 120 |  | 161 | 62 | 112 |
|  | 170 | 95 | 125 |  | 142 | 61 | 105 |
|  | 168 | 88 | 119 |  | 139 | 81 | 107 |
|  | 172 | 97 | 123 |  | 139 | 75 | 93 |

**Table S5: Raw data of the optical density of GFAP and Iba-1/0.01mm^2^ in striatum**

|  | control | QA+PBS | QA+Ipsc |  | control | QA+PBS | QA+Ipsc |
| --- | --- | --- | --- | --- | --- | --- | --- |
| GFAP | 24379 | 27937 | 30181 | Iba-1 | 18231 | 25923 | 29044 |
|  | 26548 | 28500 | 30870 |  | 18309 | 25738 | 31890 |
|  | 24768 | 27489 | 35001 |  | 19467 | 24628 | 30490 |
|  | 24098 | 26349 | 31827 |  | 21408 | 24390 | 35574 |
|  | 24908 | 25981 | 29193 |  | 21548 | 23589 | 35458 |
|  | 24789 | 25939 | 31591 |  | 19308 | 23719 | 32429 |
|  | 26153 | 26018 | 30798 |  | 19521 | 25490 | 32402 |
|  | 25883 | 27147 | 34178 |  | 19018 | 25098 | 33139 |
|  | 26594 | 27409 | 32085 |  | 18702 | 24902 | 31204 |
|  | 26438 | 29267 | 32121 |  | 18671 | 26133 | 30904 |
|  | 24090 | 28071 | 32512 |  | 16302 | 23963 | 28804 |
|  | 23518 | 26798 | 30553 |  | 19301 | 27513 | 31259 |
|  | 23239 | 26371 | 33020 |  | 20993 | 23972 | 32502 |
|  | 23449 | 28113 | 34030 |  | 22933 | 23019 | 32194 |
|  | 24009 | 27880 | 31904 |  | 19037 | 23984 | 35500 |
|  | 24382 | 28001 | 28894 |  | 18290 | 27089 | 33295 |
|  | 23003 | 25790 | 34511 |  | 19373 | 27088 | 28840 |
|  | 23398 | 26002 | 32294 |  | 19496 | 26228 | 33920 |
|  | 25778 | 26988 | 32091 |  | 18193 | 25981 | 31993 |
|  | 26875 | 25982 | 32678 |  | 18550 | 23789 | 29605 |
|  | 23517 | 25499 | 33127 |  | 19030 | 25818 | 33291 |
|  | 24319 | 26780 | 32561 |  | 18156 | 23448 | 32058 |
|  | 25098 | 26153 | 32841 |  | 19039 | 27309 | 34628 |
|  | 23678 | 27056 | 33087 |  | 21794 | 26202 | 35205 |
|  | 24409 | 28956 | 33072 |  | 20284 | 25091 | 33950 |
|  | 24304 | 29057 | 31034 |  | 21395 | 25838 | 29651 |
|  | 23432 | 25689 | 30529 |  | 18856 | 24621 | 32405 |
|  | 24130 | 27493 | 29901 |  | 18159 | 24013 | 29652 |
|  | 26165 | 26539 | 32561 |  | 21234 | 27380 | 29401 |
|  | 22858 | 27812 | 30181 |  | 21549 | 23244 | 32576 |
|  | 25090 | 27098 | 31910 |  | 18034 | 23180 | 33571 |
|  | 22640 | 25298 | 30768 |  | 18423 | 26002 | 31749 |

**Table S6: Raw data of the optical density of Western blot**

|  | control | QA+PBS | QA+IPSC |
| --- | --- | --- | --- |
| NEUN | 4001304 | 2458152 | 2937039 |
|  | 3128050 | 2263486 | 2856019 |
|  | 3789034 | 2298523 | 2253980 |
| D32 | 1011183 | 815165 | 956774 |
|  | 1236851 | 688208 | 919125 |
|  | 1109287 | 768210 | 923109 |
| GFAP | 528911 | 591711 | 921947 |
|  | 529752 | 901118 | 1057421 |
|  | 540982 | 782098 | 998209 |
| IBA | 633285 | 893098 | 969021 |
|  | 659532 | 930982 | 820981 |
|  | 678929 | 799819 | 810982 |
| ACTIN | 326759 | 341201 | 321982 |
|  | 330982 | 320198 | 312890 |
|  | 341340 | 310982 | 332785 |
